# Detection of unknown atrial fibrillation by prolonged ECG monitoring in an all-comer patient cohort and association with clinical and Holter variables

**DOI:** 10.1136/openhrt-2019-001151

**Published:** 2020-05-04

**Authors:** Muhammad Jawad-Ul-Qamar, Winnie Chua, Yanish Purmah, Mohammad Nawaz, Chetan Varma, Russell Davis, Abdul Maher, Larissa Fabritz, Paulus Kirchhof

**Affiliations:** 1 Institute of Cardiovascular Sciences, University of Birmingham, Birmingham, UK; 2 Department of Cardiology, Sandwell and West Birmingham Hospitals NHS Trust, West Bromwich, UK; 3 University Heart and Vascular Center UKE Hamburg, Hamburg, Germany

**Keywords:** atrial fibrillation, stroke, Holter ECG

## Abstract

**Objectives:**

Prolonged ECG monitoring is clinically useful to detect unknown atrial fibrillation (AF) in stroke survivors. The diagnostic yield of prolonged ECG monitoring in other patient populations is less well characterised. We therefore studied the diagnostic yield of prolonged Holter ECG monitoring for AF in an *unselected* patient cohort referred from primary care or seen in a teaching hospital.

**Methods:**

We analysed consecutive 7-day ECG recordings in unselected patients referred from different medical specialities and assessed AF detection rates by indication, age and comorbidities.

**Results:**

Seven-day Holter ECGs (median monitoring 127.5 hours, IQR 116 to 152) were recorded in 476 patients (mean age 54.6 (SD 17.0) years, 55.9% female) without previously known AF, requested to evaluate palpitations (n=241), syncope (n=99), stroke or transient ischaemic attack (n=75), dizziness (n=29) or episodic chest pain (n=32). AF was newly detected in 42/476 (8.8%) patients. Oral anticoagulation was initiated in 40/42 (95.2%) patients with newly detected AF. Multivariate logistic regression, adjusted for age, sex and monitoring duration found four clinical parameters to be associated with newly detected AF: hypertension OR=2.54, (1.08 to 8.61) (adjusted OR (95% CI)), p=0.034; previous stroke or TIA OR=4.14 (1.81 to 13.01), p=0.001; left-sided valvular heart disease OR=5.07 (2.48 to 18.70), p<0.001 and palpitations OR=2.86, (1.33 to 10.44), p=0.015.

**Conclusions:**

Open multispeciality access to prolonged ECG monitoring, for example, as part of integrated, cross-sector AF care, can accelerate diagnosis of AF and increase adequate use of oral anticoagulation, especially in older and symptomatic patients with comorbidities.

**Key questions**

**What is already known about this subject?**

- Systematic ECG monitoring is recommended for detection of atrial fibrillation (AF) in patients with ischaemic stroke.

**What does this study add?**

- Detection of AF through in an all-comer patient population through systematic ECG monitoring is not previously reported.

**How might this impact on clinical practice?**

- Judicious use of Holter ECG monitoring for new AF detection especially in the presence of clinical factors increasing the chances of AF detection could have significant diagnostic and therapeutic implications.

## Introduction

Atrial fibrillation (AF), a common cause of stroke and cardiovascular death, often remains undetected until a severe complication occurs, such as an ischaemic stroke.^1 2^ Timely diagnosis of AF is a major clinical need with immediate therapeutic consequences in patients at high stroke risk who would benefit from oral anticoagulation.^3 4^ Systematic ECG monitoring improves detection of undiagnosed paroxysmal AF in survivors of a stroke or transient ischaemic attack (TIA), detecting AF in around 4% of unselected stroke survivors using 3-day Holter monitoring^5 6^ and in up to 25% of selected patients with cryptogenic stroke using implantable monitors.^3 7^ Recent guidelines recommend at least 72 hours of ECG monitoring in stroke survivors to detect silent AF.^1 8^ The diagnostic yield of prolonged ECG monitoring in other groups of patients at risk of AF has not been studied systematically. We therefore quantified AF detection rates of 7-day Holter ECG monitoring in an unselected cohort of consecutive patients referred for such monitoring, and identified factors that are associated with detection of unknown AF by multivariate regression.

## Methods

### Patients and procedures

**Figure 1:**
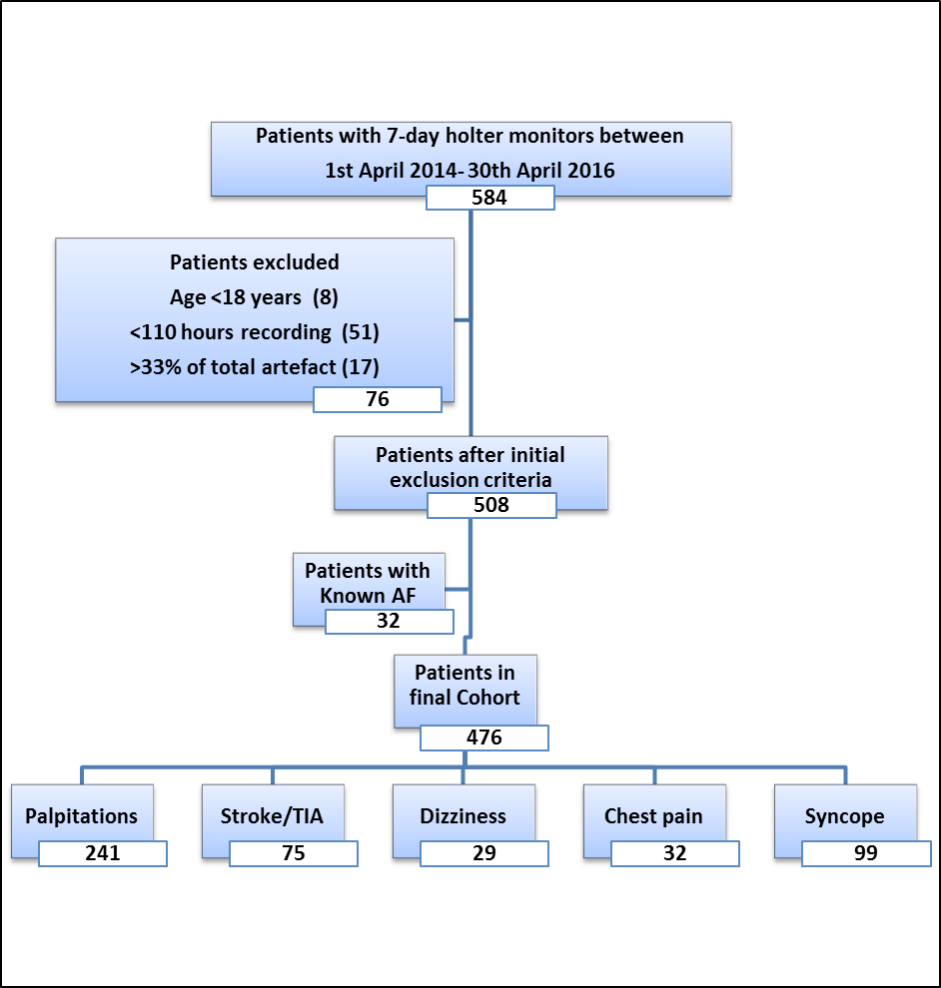
Overview of the patient population. AF, atrial fibrillation; TIA, transient ischaemic attack.

We analysed 584 consecutive 7-day Holter ECG recordings performed in adult patients (>18 years) in Sandwell and West Birmingham Hospitals (SWBH) NHS Trust from 1^st^ April 2014 to 30^th^ April 2016. Open access 7-day Holter monitoring is available to hospital physicians and general practitioners in participating practices in our region for various indications. All recordings made through Spacelabs Healthcare modular digital Holter recorder, with Lifecard CF, were initially analysed and reported by trained cardiac physiologists using Pathfinder 1.71 running on Sentinel server V.17.1.1 (Spacelabs, UK). All analyses were subsequently reviewed by two cardiology physicians (JQ, YP). We did not find any discrepancy between the initial analysis and its review for AF detection and any Holter ECG parameter of interest. We excluded 108 patients with known AF, those <18 years of age and recordings with <110 hours analysable monitoring duration. The remaining 476 recordings constituted our primary analysis data set (figure 1). Clinical information was verified by three researchers (JQ, YP, MN) from hospital records. This analysis was approved by the clinical effectiveness department at SWBH NHS Trust as part of our ongoing quality improvement programme (QIP SG323).

### Definitions

The study outcome, AF, was defined as chaotic atrial arrhythmia with loss of p wave activity and irregular RR interval lasting for at least 30 s. We defined *atrial or supraventricular ectopic* as narrow QRS complex similar to sinus beat with different or absent p wave and <80% of prevailing RR interval. *Supraventricular ectopic (SVE) runs* were defined as regular or irregular runs of three or more consecutive atrial ectopics lasting for less than 30 s. *Sinus pause* was defined as increase in RR interval due to delay in p wave activity resulting in a pause >2 s. We categorised significant atrial ectopy as ≥50 atrial ectopics/day and significant sinus pauses as ≥10 sinus pauses/day based on cut-offs calculated through receiver operating characteristic (ROC) curve. All comorbidities were taken as previously documented through records from primary care or specialist clinics. Valvular disease was taken as moderate-to-severe left (mitral or aortic) valvular disease, stenosis or regurgitation confirmed on prior echocardiography.

### Statistical analysis

Normality was assessed by inspecting the frequency distribution curves. For normally distributed variables we reported mean and SD, for other parameters median and IQR. Categorical variables were compared using χ^2^ tests and continuous variables using independent t-test or Mann-Whitney U test as appropriate. The p values of <0.05 were considered statistically significant. The cut-offs for continuous variables were calculated through the Youden's index (maximum sensitivity and minimum 1-specificity) obtained from the receiver operating characteristics (ROC) curve. The univariate relationship to the outcome was quantified using logistic regression. A multivariate logistic regression model through enter method was fitted using a combination of various clinical variables which includes hypertension, left-sided moderate/severe valvular disease, previous cerebrovascular accident and palpitations which were chosen due to their clinical relevance. The derived model was adjusted for sex, age and duration of recording. The model was internally validated using bootstrapping to produce an optimism-adjusted model. We assessed the bootstrapped model’s performance by quantifying the C-statistic (equivalent to area under the ROC curve). All the statistical analyses were performed using SPSS V.24 (IBM).

## Results

### Clinical characteristics of the patient population and AF detection

Mean age of the study population was 56 years, 56% were female, and 40% were older than 60 years (table 1). AF was diagnosed in 42/476 patients (8.8%) of which three had new AF throughout the recording and the remaining had paroxysmal AF. Median number of AF episodes was 4 (IQR 1.75 to 5.25), total duration of longest AF episode was 28 min (IQR 7.9 to 180.4) and median duration of longest episode was 731 min (IQR 3.9 to 115.1), respectively. After detection of AF, 40/42 (95.2%) patients were started on oral anticoagulation.

**Table 1:** Baseline characteristics, comorbidities and Holter parameters

Variable	Total (n=476)	AF detected (n=42)	AF not detected (n=434)	p value
Clinical parameters				
Age, years, mean (SD)	56.4 (17.0)	69.8 (12.2)	53.1 (16.7)	**<0.001**
Female, n (%)	266 (56)	22 (52)	244 (56)	0.632
Hypertension, n (%)	173 (36.3)	32 (76.1)	141 (32.4)	**<0.001**
Diabetes, n (%)	78 (16.3)	11 (26.2)	67 (18)	0.072
Coronary artery disease, n (%)	102 (21.4)	17 (40.4)	85 (19.5)	**0.002**
Hypercholesterolaemia, n (%)	177 (37.1)	17 (40.4)	160 (36.8)	0.644
History of stroke or transient ischaemic attack, n (%)	111 (23.3)	23 (54.7)	88 (20.2)	**<0.001**
Valvular disease, n (%)	72 (15.1)	18 (42.8)	54 (12.4)	**<0.001**
Palpitations, n (%)	241 (50.63)	32 (76.19)	209 (48.15)	**0.001**
Holter parameters				
Recording, hours, median (IQR)	127.5 (116 to 152)	153.4 (133.7 to 166.0)	124.0 (115.0 to 127.5)	**0.001**
Mean heart rate, bpm, mean (SD)	79.9 (13.3)	104.7 (13.0)	77.5 (11.2)	**0.001**
Max heart rate, bpm, mean (SD)	139.1 (18.2)	144.9 (12.4)	138.5 (18.6)	**0.004**
Min heart rate, bpm, mean (SD)	49.2 (8.3)	50.4 (10.5)	49.0 (8.1)	0.417
≥10 sinus pauses/day (%)	77 (16.1)	26 (61.9)	51 (11.7)	**<0.001**
≥50 atrial ectopics/day (%)	156 (32.7)	36 (85.7)	120 (27.6)	**<0.001**
≥500 ventricular ectopics/recording (%)	443 (93)	42 (100)	401 (92.3)	0.064
Supraventricular ectopic (SVE) runs, (%)	70 (14.7)	26 (61.9)	44 (10.1)	<**0.001**
Longest atrial run, mean (SD)	8.7 (4.0)	10.9 (2.8)	8.1 (4.0)	**<0.001**

Data presented as number, n(percentage,%) for categorical variables, mean (SD) for parametric continuous variables, and median (IQR) for non-parametric continuous variables. p value is significant if < 0.05.

AF, atrial fibrillation; bpm, beats per minute; SD, Standard Deviation.

**Figure 2:**
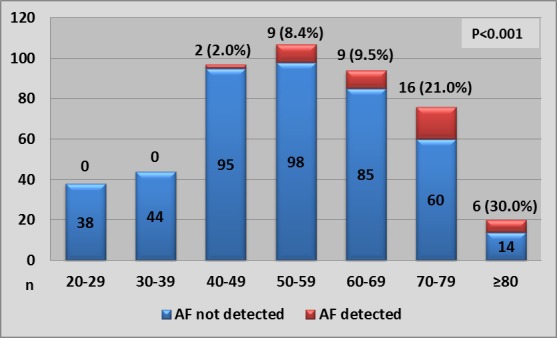
Number of patients with and without newly detected AF split by age deciles. Percentage of patients with newly detected AF is given above the bars for each decile. AF, atrial fibrillation.

**Figure 3:**
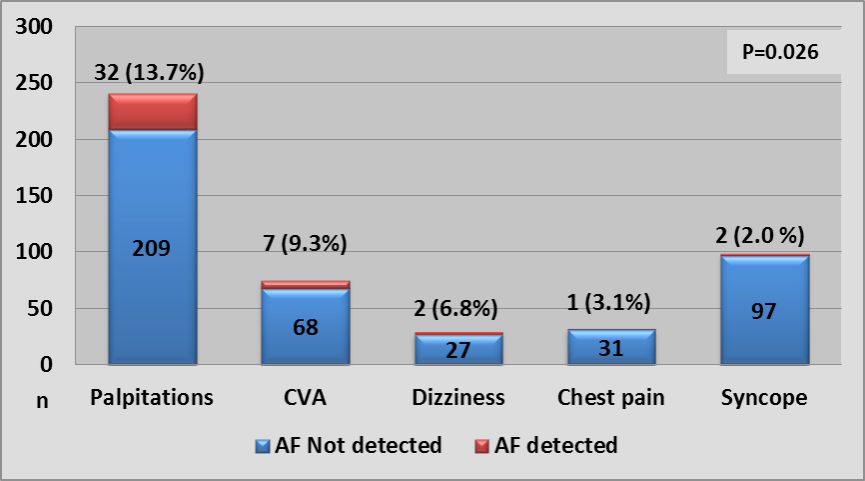
Number of patients with and without newly detected AF by underlying conditions to justify the 7-day Holter monitor. AF, atrial fibrillation; CVA, cerebrovascular accident.

Patients with AF detected were older (mean 69.8 years, SD 69.8) with no significant difference in sex and 31/42 (73.8%) patients with AF were age 60 years or more (figure 2). The AF group had more comorbidities such as hypertension, coronary artery disease, history of stroke or transient ischaemic attack, moderate-to-severe left-sided valvular disease and palpitations. There was no difference seen for diabetes and hypercholesterolaemia. The recording duration in AF group was longer, median (IQR), 153.4 hours (133.7 to 166.0) versus non-AF group 124.0 hours (115.0 to 127.5). Moreover there were more sinus pauses, atrial ectopics and SVE runs. If present, SVE runs were longer in the AF group. AF was more often found in stroke survivors (7/75, 9.3%) and in patients with palpitations (32/241, 13.3%) than in patients with dizziness (2/29, 6.8%), syncope (2/99, 2%) or chest pain (1/32, 3.1%) (figure 3). The source of referral for the test did not affect AF detection (cardiology 8.7% (19/218), general medicine 9% (19/222) and primary care 8.6% (4/46)).

### Other relevant findings

Other medically relevant findings were second degree atrioventricular (AV) block (10/474, 2.1%), third degree AV block (2/474, 0.4%) and non-sustained ventricular tachycardia (12/474, 2.5%). These were all detected in patients who underwent monitoring for dizziness. Artefact burden in the final cohort was 3.3% (SD 3.5)

### Clinical parameters associated with AF

Univariately, AF was more likely to be detected in patients who are older (OR 10.85, 95% CI 10.56 to 11.15 for each 10 year increase in age), and in those presenting with palpitations (OR 3.44, 95% CI 1.65 to 7.18, p=0.001), have hypertension (OR 6.65, 95% CI 3.17 to 13.90, p<0.001), coronary artery disease (OR 2.79, 95% CI 1.44 to 5.40, p=0.003), history of ischaemic strokes or TIA (OR 4.76, 95% CI 2.48 to 9.12, p<0.001), or moderate-to-severe left-sided valve disease (OR 5.27, 95% CI 2.68 to 10.35, p<0.001).

### Holter parameters associated with AF

AF detection was also univariately associated with longer ECG monitoring (OR 1.05, 95% CI 1.03 to 1.07 for each additional hour of monitoring), sinus pauses (OR 12.2, 95% CI 6.13 to 24.27, p<0.001), significant atrial ectopics (OR 15.70, 95% CI 6.45 to 38.21, p<0.001) and presence of SVE runs (OR 14.4, 95% CI 7.17 to 28.90, p<0.001, table 2).

**Table 2:** Factors associated with unknown AF

Variables	OR	95% Confidence Intervals (CI)		p value
		Lower limit	Upper limit	
Univariate logistic regression analysis				
Age (per year increase)	1.08	1.06	1.11	**<0.001**
Hypertension	6.65	3.17	13.90	**<0.001**
Diabetes	1.94	0.93	4.07	0.077
Coronary artery disease	2.79	1.44	5.40	**0.002**
Hypercholesterolaemia	1.16	0.61	2.22	0.644
Valvular disease	5.27	2.68	10.35	**<0.001**
Previous CVA/TIA	4.76	2.48	9.12	**<0.001**
Palpitations	3.44	1.65	7.18	**0.001**
Recording duration (per hour increase)	1.05	1.03	1.07	**<0.001**
≥10 sinus pauses/day	12.2	6.13	24.27	**<0.001**
≥50 AE/day	15.70	6.45	38.21	**<0.001**
Presence of SVE runs	14.40	7.17	28.90	**<0.001**
**Variables (bootstrapped)**				
Multivariate logistic regression analysis				
Hypertension	2.54	1.08	8.61	**0.034**
Previous CVA	4.14	1.81	13.01	**0.001**
Valvular disease	5.07	2.48	18.70	**<0.001**
Palpitations	2.86	1.33	10.44	**0.015**

AE, atrial ectopic; AF, atrial fibrillation; CVA, cerebrovascular accident; SAE, significant atrial ectopy; SVE, supraventricular ectopic; TIA, transient ischaemic attack.

**Figure 4:**
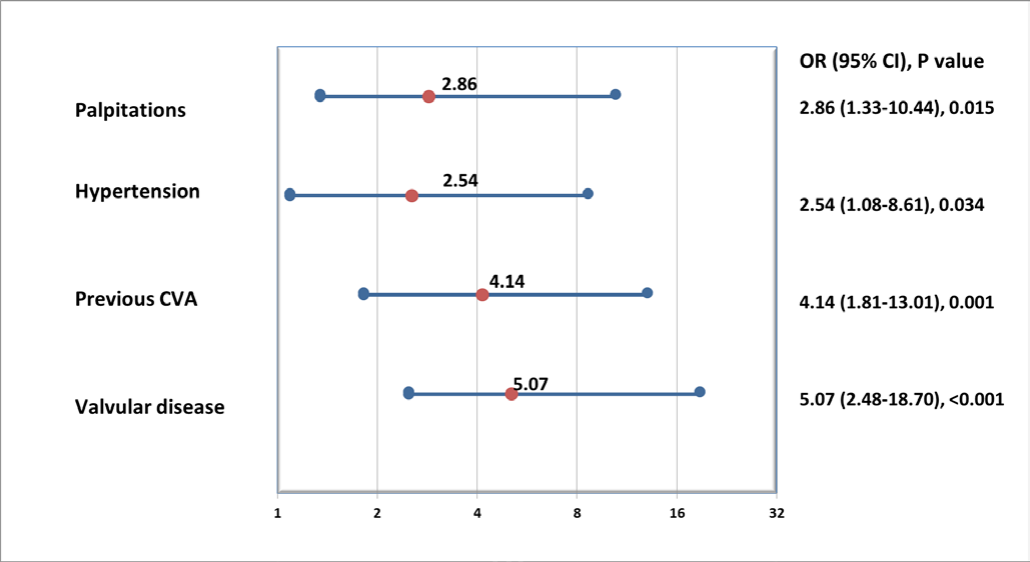
Individual predictors of newly detected AF by 7-day Holter, shown as hazard ratio and 95% confidence interval. CVA, cerebrovascular accident.

Multivariate regression, performed through enter method identified four variables to be significantly associated with undetected AF: Previous stroke or transient ischaemic attack bootstrapped OR=4.14 (95% CI 1.81 to 13.01), p=0.001; left-sided moderate-to-severe valvular disease OR=5.07 (95% CI 2.48 to 18.70), p<0.001; palpitations OR=2.86, (95% CI 1.33 to 10.44), p=0.015 and hypertension OR=2.54, (95% CI 1.08 to 8.61) p=0.034; table 2, figure 4. The model was adjusted for age, sex and duration of recording.

**Figure 5:**
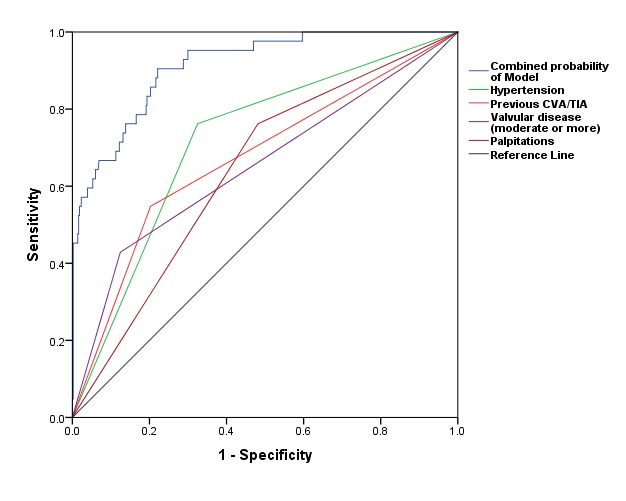
Receiver operating characteristic (ROC) curves for the different AF prediction models. See text for details.CVA, cerebrovascular accident; TIA, transient ischaemic attack.

The area under the curve (C statistic) for our multivariate model was 0.91 (95% CI 0.87 to 0.95) with individual C-statistics for each variable mentioned in figure 5, table 3.

**Table 3:** Area under the curve values and CIs

Variable(s)	Area	95% Confidence Intervals (CI)		p value
		Lower bound	Upper bound	
Combined model	0.912	0.870	0.954	**<0.001**
Hypertension	0.719	0.639	0.798	**<0.001**
Previous CVA	0.672	0.580	0.765	**<0.001**
Valvular disease	0.652	0.555	0.749	**<0.001**
Palpitations	0.640	0.558	0.723	**0.003**

CVA, cerebrovascular accident.

## Discussion

We found that 7-day ECG monitoring detected unknown AF in 9% of an unselected cohort presenting with various symptoms. Almost all patients were initiated on oral anticoagulation following AF detection. The AF detection rates of 9.3% in stroke survivors is comparable to AF rates in IDEAS (4.3%, 72 hours monitoring,^5^ FIND-AF (12.5%, 10 days monitoring)^9^ and FIND-AF_randomised_ (14%).^6^ The data supports the wider use of systematic ECG monitoring to allow early initiation of therapy for AF, particularly stroke prevention, for example, as part of an integrated approach to AF care. External validation of our findings is desirable.

### Factors associated with newly detected AF

Patients with AF were older (table 1, figures 2 and 4) than those without AF, similar to other reports^10 11^ and consistent with the known finding that older age is associated with prevalent AF.^12 13^ We found that each decade increase in age increased the odds of detecting AF by 10.8. In addition, previous history of stroke and left-sided valvular heart disease was associated with silent AF in univariate analysis which is comparable to prior reports.^14 15^ In our study palpitations were the symptom that was most frequently associated with underlying AF (figure 4). Our analysis supports some prior reports associating palpitations with AF^16^ while others found a more ephemeral nature of presenting symptoms.^17^ As expected, longer monitoring duration increased AF detection.^18^ In addition, excessive supraventricular ectopic activity was associated with unknown AF (table 2, figure 4), comparable to prior analyses.^19–21^ Sinus node disease, identified by sinus pauses,^22^ has been linked to underlying AF.^23 24^ Although, sinus pauses were not part of our multivariate model, but there is evidence to suggest that it could be associated with unknown AF, confirming findings from one prior study.^25^ We suggest further ECG monitoring, beyond 7 days, to look for AF in clinically high risk patients as identified by our multivariate model, especially, if they have evidence of significant atrial ectopy or sinus pauses on prior monitoring.

### Open access ECG monitoring - an alternative approach to increase detection of unknown AF?

Current guidelines recommend pulse palpation followed by an ECG in patients with an irregular pulse in populations >65 years to detect AF.^1 8^ Pulse palpation can be replaced by a blood pressure machine.^26 27^ Systematic prolonged ECG monitoring is recommended in stroke survivors. In addition, community-based ECG screening programmes using patient-operated devices are able to identify patients with silent AF, especially when elderly populations are screened, for example, those aged 75 years or more.^28–30^ Such population-based screening programmes require a specific infrastructure that is currently confined to a research environment or not available at all. Our study suggests that open access to prolonged ECG monitoring has a high diagnostic yield in patients with various symptoms, opening up an alternative approach to increase the detection of unknown AF. Open access to ECG monitoring has the advantage of using existing health infrastructure, but is limited to populations with symptoms.

### Limitations

Our findings will need validation in other, external patient cohorts. Although the number of patients in our study was reasonable for a busy teaching hospital however more patients are required for further studies especially to assess Holter ECG variables with incident AF. The number of patients also had an effect on the relatively wide CIs for the calculated ORs. AF group had longer duration of monitoring that could introduce bias. To rectify this we adjusted our multivariate variable for duration of recording. Moreover, older patient usually have higher incidence of comorbidities which may be one reason why the multivariate model strongly predicted AF.

## Conclusion

Seven-day Holter ECG monitoring detects unknown AF in around 9% of patients with various symptoms in an open access, ‘all-comer’ setting, suggesting that this is a useful diagnostic approach to enhance detection of unknown AF. Risk factor analysis for paroxysmal AF and systematic evaluations of treatment and outcomes after AF screening are needed to define populations for screening silent AF. Comparable AF detection rates between cardiology and primary care also supports provision of ECG monitoring using open-access services across various healthcare sectors.

## References

[1] Freedman B, Camm J, Calkins H, et al Screening for atrial fibrillation: a report of the AF-SCREEN international collaboration. Circulation 2017;135:1851–67. 10.1161/CIRCULATIONAHA.116.026693 28483832

[2] Kirchhof P, Breithardt G, Bax J, et al A roadmap to improve the quality of atrial fibrillation management: proceedings from the fifth atrial fibrillation Network/European heart rhythm association consensus conference. Europace 2016;18:37–50. 10.1093/europace/euv304 26481149

[3] Gladstone DJ, Spring M, Dorian P, et al Atrial fibrillation in patients with cryptogenic stroke. N Engl J Med 2014;370:2467–77. 10.1056/NEJMoa1311376 24963566

[4] Hart RG, Pearce LA, Aguilar MI Meta-analysis: antithrombotic therapy to prevent stroke in patients who have nonvalvular atrial fibrillation. Ann Intern Med 2007;146:857–67. 10.7326/0003-4819-146-12-200706190-00007 17577005

[5] Grond M, Jauss M, Hamann G, et al Improved detection of silent atrial fibrillation using 72-hour Holter ECG in patients with ischemic stroke: a prospective multicenter cohort study. Stroke 2013;44:3357–64. 10.1161/STROKEAHA.113.001884 24130137

[6] Wachter R, Gröschel K, Gelbrich G, et al Holter-electrocardiogram-monitoring in patients with acute ischaemic stroke (Find-AF_RANDOMISED_): an open-label _randomised_ controlled trial. Lancet Neurol 2017;16:282–90. 10.1016/S1474-4422(17)30002-9 28187920

[7] Sanna T, Diener H-C, Passman RS, et al Cryptogenic stroke and underlying atrial fibrillation. N Engl J Med 2014;370:2478–86. 10.1056/NEJMoa1313600 24963567

[8] Kirchhof P, Benussi S, Kotecha D, et al 2016 ESC guidelines for the management of atrial fibrillation developed in collaboration with EACTS. Eur Heart J 2016;37:2893–962. 10.1093/eurheartj/ehw210 27567408

[9] Stahrenberg R, Weber-Krüger M, Seegers J, et al Enhanced detection of paroxysmal atrial fibrillation by early and prolonged continuous Holter monitoring in patients with cerebral ischemia presenting in sinus rhythm. Stroke 2010;41:2884–8. 10.1161/STROKEAHA.110.591958 20966415

[10] Suissa L, Bertora D, Lachaud S, et al Score for the targeting of atrial fibrillation (STAF): a new approach to the detection of atrial fibrillation in the secondary prevention of ischemic stroke. Stroke 2009;40:2866–8. 10.1161/STROKEAHA.109.552679 19461041

[11] Verdecchia P, Reboldi G, Gattobigio R, et al Atrial fibrillation in hypertension: predictors and outcome. Hypertension 2003;41:218–23. 10.1161/01.hyp.0000052830.02773.e4 12574085

[12] Leitch JW, Thomson D, Baird DK, et al The importance of age as a predictor of atrial fibrillation and flutter after coronary artery bypass grafting. J Thorac Cardiovasc Surg 1990;100:338–42. 10.1016/S0022-5223(19)35525-4 2391970

[13] Aranki SF, Shaw DP, Adams DH, et al Predictors of atrial fibrillation after coronary artery surgery. Current trends and impact on hospital resources. Circulation 1996;94:390–7. 10.1161/01.cir.94.3.390 8759081

[14] Poli S, Diedler J, Härtig F, et al Insertable cardiac monitors after cryptogenic stroke - a risk factor based approach to enhance the detection rate for paroxysmal atrial fibrillation. Eur J Neurol 2016;23:375–81. 10.1111/ene.12843 26470854

[15] Christensen LM, Krieger DW, Højberg S, et al Paroxysmal atrial fibrillation occurs often in cryptogenic ischaemic stroke. Final results from the SURPRISE study. Eur J Neurol 2014;21:884–9. 10.1111/ene.12400 24628954

[16] Hansson A, Madsen-Härdig B, Olsson SB Arrhythmia-provoking factors and symptoms at the onset of paroxysmal atrial fibrillation: a study based on interviews with 100 patients seeking Hospital assistance. BMC Cardiovasc Disord 2004;4:13. 10.1186/1471-2261-4-13 15291967 PMC514544

[17] Smeets JLRM Paroxysmal atrial fibrillation: why patients experience different symptoms from the same arrhythmia? Neth J Med 2005;63:154–5. 15952482

[18] Kirchhof P, Auricchio A, Bax J, et al Outcome parameters for trials in atrial fibrillation: Executive summary: recommendations from a consensus conference organized by the German atrial fibrillation competence network (AFNET) and the European heart rhythm association (EHRA). Eur Heart J 2007;28:2803–17. 10.1093/eurheartj/ehm358 17897924

[19] Waktare JE, Hnatkova K, Sopher SM, et al The role of atrial ectopics in initiating paroxysmal atrial fibrillation. Eur Heart J 2001;22:333–9. 10.1053/euhj.2000.2517 11161952

[20] Perez MV, Dewey FE, Marcus R, et al Electrocardiographic predictors of atrial fibrillation. Am Heart J 2009;158:622–8. 10.1016/j.ahj.2009.08.002 19781423

[21] Weber-Krüger M, Gröschel K, Mende M, et al Excessive supraventricular ectopic activity is indicative of paroxysmal atrial fibrillation in patients with cerebral ischemia. PLoS One 2013;8:e67602. 10.1371/journal.pone.0067602 23840747 PMC3695922

[22] Rodriguez RD, Schocken DD Update on sick sinus syndrome, a cardiac disorder of aging. Geriatrics 1990;45:26–36. 2403955

[23] Alt E, Lehmann G Stroke and atrial fibrillation in sick sinus syndrome. Heart 1997;77:495–7. 10.1136/hrt.77.6.495 9227288 PMC484787

[24] Zdrojewicz Z, Iwankiewicz G, Rutkowski J, et al [Paroxysmal atrial fibrillation as an integral part of sick (damaged) sinus syndrome]. Wiad Lek 1989;42:13–19. 2781799

[25] Soeki T, Matsuura T, Tobiume T, et al Clinical, electrocardiographic, and echocardiographic parameter combination predicts the onset of atrial fibrillation. Circ J 2018;82:2253–8. 10.1253/circj.CJ-17-0758 29848884

[26] Chan P-H, Wong C-K, Pun L, et al Head-To-Head comparison of the AliveCor heart monitor and Microlife WatchBP office AFIB for atrial fibrillation screening in a primary care setting. Circulation 2017;135:110–2. 10.1161/CIRCULATIONAHA.116.024439 28028066

[27] Chan P-H, Wong C-K, Pun L, et al Diagnostic performance of an automatic blood pressure measurement device, Microlife WatchBP home a, for atrial fibrillation screening in a real-world primary care setting. BMJ Open 2017;7:e013685. 10.1136/bmjopen-2016-013685 PMC5577883 28619766

[28] Svennberg E, Engdahl J, Al-Khalili F, et al Mass screening for untreated atrial fibrillation: the STROKESTOP study. Circulation 2015;131:2176–84. 10.1161/CIRCULATIONAHA.114.014343 25910800

[29] Engdahl J, Svennberg E, Friberg L, et al Stepwise mass screening for atrial fibrillation using N-terminal pro B-type natriuretic peptide: the STROKESTOP II study design. Europace 2017;19:297–302. 10.1093/europace/euw319 28011798

[30] Chan N-Y, Choy C-C Screening for atrial fibrillation in 13 122 Hong Kong citizens with smartphone electrocardiogram. Heart 2017;103:24–31. 10.1136/heartjnl-2016-309993 27733533

